# Lysophosphatidic Acid Receptor 5 Plays a Pathogenic Role in Brain Damage after Focal Cerebral Ischemia by Modulating Neuroinflammatory Responses

**DOI:** 10.3390/cells9061446

**Published:** 2020-06-10

**Authors:** Arjun Sapkota, Chi-Ho Lee, Se Jin Park, Ji Woong Choi

**Affiliations:** 1 College of Pharmacy, Gachon University, Incheon 21936, Korea; sapkotaa07@gmail.com (A.S.); lch7835@nate.com (C.-H.L.); 2 School of Natural Resources and Environmental Sciences, Kangwon National University, Chuncheon 24341, Korea

**Keywords:** cerebral ischemia, LPA_5_, TCLPA5, Microglial activation

## Abstract

Receptor-mediated lysophosphatidic acid (LPA) signaling has come to be considered an important event for various diseases. In cerebral ischemia, LPA_1_ has recently been identified as a receptor subtype that mediates brain injury, but the roles of other LPA receptor subtypes remain unknown. Here, we investigated the potential role of LPA_5_ as a novel pathogenic factor for cerebral ischemia using a mouse model of transient middle cerebral artery occlusion (tMCAO). LPA_5_ was upregulated in the ischemic core region after tMCAO challenge, particularly in activated microglia. When TCLPA5, a selective LPA_5_ antagonist, was given to tMCAO-challenged mice immediately after reperfusion, brain damage, including brain infarction, functional neurological deficit, and neuronal and non-neuronal apoptosis, was reduced in mice. Similarly, delayed TCLPA5 administration (at three hours after reperfusion) reduced brain infarction and neurological deficit. The histological results demonstrated that TCLPA5 administration attenuated microglial activation, as evidenced by the decreased Iba1 immunoreactivities, the number of amoeboid cells, and proliferation in an injured brain. TCLPA5 administration also attenuated the upregulation of the expression of pro-inflammatory cytokines at mRNA levels in post-ischemic brain, which was also observed in lipopolysaccharide-stimulated BV2 microglia upon LPA_5_ knockdown. Overall, this study identifies LPA_5_ as a novel pathogenic factor for cerebral ischemia, further implicating it as a promising target for drug development to treat this disease.

## 1. Introduction

Cerebral ischemia is a type of neurological disease caused by a sudden blockade of blood oxygen and glucose supply in the brain. It is one of the leading causes of death and disability globally, annually affecting 13.7 million people and causing 5.5 million deaths [1]. Treatment options for cerebral ischemia are limited because tissue plasminogen activator (tPA) and thrombectomy are commonly-used treatments for acute ischemic stroke [2,3]. To date, numerous efforts have been made to develop new therapeutics that modulate the pathogenesis of cerebral ischemia, including glutamate-mediated excitotoxicity, oxidative stress, and neuroinflammatory responses [4,5,6]. Based on this, many candidates have been suggested as therapeutic targets [7,8]. As these studies have not led to the successful development of a new drug, it remains important to identify a novel pathogenic factor for cerebral ischemia.

Lysophosphatidic acid (LPA), an important bioactive lysophospholipid, is present in blood and various tissues [9]. LPA is associated with different biological functions in human body systems through the activation of its specific G protein-coupled receptor subtypes, LPA_1–6_ [9,10]. The specific biological roles of LPA receptors in various diseases have been examined through gain- and loss-of-function studies of each receptor subtype [9,11]. In the central nervous system (CNS), LPA receptors are expressed on different cell types [10,11,12], and they play critical roles in CNS disorders, including spinal cord injury [13,14], sepsis [15], neuropathic pain [16,17], fetal hydrocephalus [18], and schizophrenia [19]. Even in cerebral ischemia, LPA receptors have been suggested to contribute to brain injury after ischemic challenge. Amounts of LPA, a ligand for LPA receptors, have been reported to be elevated in human patients with ischemic cerebrovascular disease [20] and rats with tMCAO challenges [21]. Receptor-mediated LPA signaling has also been suggested to be involved in brain damage after tMCAO challenge. In fact, previous studies have addressed the pathogenic roles of LPA_1_ in cerebral ischemia [22,23,24]. In particular, suppressing LPA_1_ activity has been shown to attenuate brain infarction, behavioral neurological deficit, and neuroinflammatory responses such as microglial activation, a common pathogenic feature of cerebral ischemia [25], in the post-ischemic brain. Other subtypes of LPA receptors besides LPA_1_ could be involved in the pathogenesis of cerebral ischemia, but further research is needed. 

LPA_5_ is a member of six LPA receptors and is structurally similar to LPA_4_ [26]. LPA_5_ is coupled with G12/13 and Gq/11 and influences various cellular events, including stress fiber formation, neurite formation, and elevated levels of intracellular calcium and cAMP [27]. LPA_5_ is expressed throughout the body including in the brain, spleen, stomach, small intestine, kidney, and skin [26,27], and some of the pathological roles of LPA_5_ have been examined in diverse pathological conditions, including itching [28], platelet activation [29], cancer [30], and pain [31,32,33,34]. Notably, LPA_5_ has recently been implicated as a critical factor to modulate microglial activation and their production of pro-inflammatory cytokines using the BV2 microglial cell line or primary murine microglia [35,36]. Furthermore, LPA_5_ has been implicated in the control of microglial phenotypes toward pro-inflammatory phenotypes by producing pro-inflammatory cytokines [35]. Since microglial activation and proinflammatory responses in the brain are important features in the pathogenesis of cerebral ischemia, these microglial biology findings strongly suggest that LPA_5_ may play an important role in the brain after ischemic challenge. 

In the current study, we have investigated the potential role of LPA_5_ as a novel pathogenic factor for cerebral ischemia using a mouse model of transient middle cerebral artery occlusion (tMCAO). We have determined whether the expression of LPA_5_ is increased in the post-ischemic brain using immunofluorescence. Next, we have determined whether LPA_5_ contributes to brain damage after tMCAO challenge using a well-known LPA_5_ antagonist, TCLPA5. We have also determined whether LPA_5_ is involved in microglial activation in the post-ischemic brain. Furthermore, we have determined the roles of LPA_5_ on neuroinflammatory responses by assessing the mRNA expression levels of pro- and anti-inflammatory cytokines in post-ischemic brain in vivo and lipopolysaccharide (LPS)-stimulated BV2 microglial cell line in vitro. 

## 2. Materials and Methods 

### 2.1. Animal

ICR male mice (six weeks old) were purchased from Orient Bio (Seongnam-Si, Korea). All animal experiments were conducted in compliance with the Institutional Animal Care and Use guidelines of the Lee Gil Ya Cancer and Diabetes Institute (LCDI) at Gachon University: LCDI-2019-0027 was approved as the animal protocol number for this study. Mice were housed with a controlled light cycle (a 12 h day/night), temperature (22 ± 2 °C), and relative humidity (60 ± 10%), and had free access to food and water ad libitum.

### 2.2. Transient Focal Cerebral Ischemia Challenge

Male ICR mice were challenged by tMCAO as previously described [24]. Briefly, mice were anesthetized with isoflurane (3%) in oxygen and nitrogen (30:70% ratio, respectively) before being kept in a supine position in an operation frame. The right common carotid artery (CCA) was separated from the vagus nerve and MCAO was induced by inserting a nylon monofilament coated with silicon (9-mm-long 5-0) from CCA bifurcation to the MCA. After ninety minutes, the monofilament was removed for reperfusion under anesthetic conditions. Body temperature was maintained at 37 °C throughout the surgery. For the sham group, mice underwent the same surgical procedure excluding MCAO.

### 2.3. TCLPA5 Administration

Mice challenged by tMCAO were randomly assigned into either the vehicle (10% cremophor EL and 10% ethanol in distilled water)- or the TCLPA5-administered group. First, different dosages of TCLPA5 (1, 3, 10, and 30 mg/kg, i.p., Tocris Bioscience, Bristol, UK) were given to mice immediately after reperfusion. To determine the effects of delayed administration, TCLPA5 was given to mice 3 h after reperfusion. The investigator was blinded to the experimental groups.

### 2.4. Determination of Functional Neurological Deficit Score and Infarction Volume

Functional neurological deficit score was obtained based on modified neurological severity score (mNSS) grade achieved in the motor, sensory, reflex, and balance tests at one or three days after tMCAO challenge, as described previously [37]. The functional neurological deficit score grades ranged from zero points for normal to eighteen points for the maximal deficit. 

At one day after tMCAO challenge, mice were sacrificed using CO_2_ exposure, and their brains were harvested to determine the infarct volume through 2% 2,3,5-triphenyltetrazolium chloride (TTC) staining, as previously described [24]. The brains were sectioned into 2-mm-thick slices and incubated with 2% TTC in saline at 37 °C. Photographs were then taken of the brain slices, and these images were used to quantify infarction volume using ImageJ software (National Institute of Mental Health, Bethesda, MD, USA). 

### 2.5. Immunohistochemical Assessment

#### 2.5.1. Tissue Preparation

Brain tissues were obtained at one or three days after tMCAO challenge. Mice were anesthetized using a mixture of Zoletil 50^®^ (10 mg/kg, i.m., Virbac Laboratories, Carros, France) and Rompun^®^ (3 mg/kg, i.m., Bayer HealthCare LLC, KS, USA), perfused with ice-cold phosphate-buffered saline (PBS), and fixed with 4% paraformaldehyde (PFA) in phosphate buffer solution. The brains were harvested, post-fixed with 4% PFA for an additional 24 h, transferred into 30% sucrose solution, and frozen in Tissue-Tek Optimal Cutting Temperature compound. The frozen brains were sectioned into 20-µm-thick coronal sections using a cryostat (RD-2230, Roundfin, Liaoning, China).

#### 2.5.2. LPA_5_ Immunofluorescence and LPA_5_/Iba1 Double Immunofluorescence

Cryostat brain sections were post-fixed with 4% PFA, treated with 0.01 M sodium citrate at 90–100 °C and 1% H_2_O_2_, and blocked with 1% fetal bovine serum (FBS) in PBS containing 0.3% Triton X-100. Sections were labeled with rabbit anti-LPA_5_ (1:200, LifeSpan BioScience, Seattle, WA, USA) overnight at 4 °C and then labeled with Cy3-conjugated secondary antibody (1:1000, Jackson ImmunoResearch, West Grove, PA, USA) for 2 h at R/T. Sections were counterstained with DAPI (Carl Roth, Karlsruhe, Germany) and mounted with VECTASHIELD^®^ (Vector Laboratories, Burlingame, CA, USA). 

For LPA_5_ and Iba1 double immunofluorescence, sections were labeled with rabbit anti-LPA_5_ and goat anti-Iba1 (1:500, Abcam, Cambridge, UK) overnight at 4 °C after blocking. These sections were then incubated with Cy3- and AF488-conjugated (1:1000, Jackson ImmunoResearch) secondary antibodies for 2 h at R/T. Sections were counterstained with DAPI and mounted with VECTASHIELD^®^.

#### 2.5.3. TUNEL Assay

Neuronal and non-neuronal apoptosis in the post-ischemic brain was analyzed through TUNEL assay. Cryostat brain sections were post-fixed with 4% PFA, treated with 50 mM ammonium chloride, exposed to 1% H_2_O_2_ in 1% NaOH, exposed with 0.03% glycine, and blocked with 1% FBS in PBS containing 0.3% Triton X-100. Sections were labeled with mouse anti-NeuN (1:200, Millipore, Burlington, MA, USA) overnight at 4 °C, then incubated with AF488-conjugated secondary antibody (1:1000, Jackson ImmunoResearch) for 2 h at R/T. Sections were washed with PBS and stained with a mixture of Enzyme solution and Label solution (In Situ Cell Death Detection Kit, Roche, Mannheim, Germany) for 1 h at R/T. These sections were washed with PBS and distilled water and then mounted with VECTASHIELD^®^.

#### 2.5.4. Iba1 Immunohistochemistry

Cryostat brain sections were treated with 1% H_2_O_2_ for oxidation, blocked with 1% FBS solution, and labeled with rabbit anti-Iba1 (1:500, Wako Pure Chemicals, Osaka, Japan) overnight at 4 °C. Sections were incubated with a biotinylated secondary antibody (1:200, Santa Cruz Biotechnology, Dallas, TX, USA) for 2 h at R/T and further incubated with ABC reagent (1:100, Vector Laboratories). These sections were developed using a DAB kit (Dako, Santa Clara, CA, USA), washed with water, dehydrated with alcohol and xylene, and mounted with Entellan media (Merck, Darmstadt, Germany).

#### 2.5.5. 5-Bromo-2′-deoxyuridine (BrdU) and Iba1 Double Immunofluorescence

Microglial proliferation was determined using double immunofluorescence against Iba1 and BrdU, as previously described [24,38]. Briefly, BrdU (50 mg/kg, i.p., Sigma-Aldrich, St. Louis, MO, USA) was administered to mice every 12 h for 2 days starting 12 h after tMCAO or sham operation. Cryostat brain sections were post-fixed with 4% PFA, treated with 2N HCl at 37 °C for DNA denaturation, neutralized with borate buffer (0.1 M, pH 8.5), blocked with 1% FBS solution, and labeled with primary antibodies rat anti-BrdU (1:200, Abcam, Cambridge, UK) and rabbit anti-Iba1(1:500, Wako Pure Chemicals) overnight at 4 °C. Sections were further labeled with secondary antibodies conjugated with Cy3 (1:1000, Jackson ImmunoResearch) and AF488 (1:1000), then mounted with VECTASHIELD^®^.

#### 2.5.6. Image Preparation and Quantification

Brain images were taken from either a bright-field and fluorescence microscope equipped with a DP72 camera (BX53T, Olympus, Japan) or a laser scanning confocal microscope (Eclipse A1 Plus, Nikon, Japan). All demonstrative photos were prepared using Adobe Photoshop Elements 8. Three different images of each brain region were used to quantify the immunopositive cells, which were then manually counted. The numbers of cells were presented as the numbers of cells per unit area. 

### 2.6. Quantitative Real-Time PCR (qRT-PCR) Analysis

Total RNA was extracted from either the ipsilateral brain at one and three days after tMCAO challenge or BV2 cells using RNAiso plus (Takara, Kusatsu, Japan). For qRT-PCR, 1 µg of total RNA was reversely transcribed to synthesize cDNA using All-in-One First-Strand cDNA Synthesis SuperMix (TransGen Biotech, Haidian, China). qRT-PCR was performed using the StepOnePlus^TM^ qRT-PCR system (Applied Biosystems, Foster City, CA, USA) with Power SYBR Green PCR master mix (Life Technologies, Carlsbad, CA, USA) and corresponding primers (primer sequences are listed in Table 1). The expression levels of the mRNAs were quantified using the 2^−ΔΔCT^ method and then normalized to β-actin.

### 2.7. Transient Transfection of LPA_5_ in BV2 Microglial Cell Line and Treatment

BV2 cells were plated onto 6-well plates (2 × 10^5^ cells/well) and transiently transfected with either LPA_5_ siRNA (Dharmacon, Lafayette, CO, USA) or non-target control siRNA (NTC siRNA) with Lipofectamine^®^ RNAiMAX reagent (Life Technologies) in serum- and antibiotic-free Dulbecco’s modified Eagle’s medium (DMEM, Life Technologies). Six hours later, the cells were recovered by incubation in DMEM containing 10% FBS, penicillin, and streptomycin for an additional two days. These cells were serum-starved overnight and stimulated with LPS (Escherichia coli serotype 026:B6, Sigma-Aldrich) for 24 h.

### 2.8. Statistical Analysis

All data were presented as mean ± S.E.M., and statistical analysis was performed using GraphPAD Prism 7 (GraphPad Software Inc., La Jolla, CA, USA). Statistical differences among the groups were analyzed by one-way ANOVA followed by Newman–Keuls test for multiple comparisons. Differences between the two groups were analyzed by Student’s *t*-test. For all analyses, statistical significance was set at *p < 0.05*.

## 3. Results

### 3.1. LPA_5_ is Upregulated in Post-Ischemic Brain, Particularly in Activated Microglia

**Figure 1:**
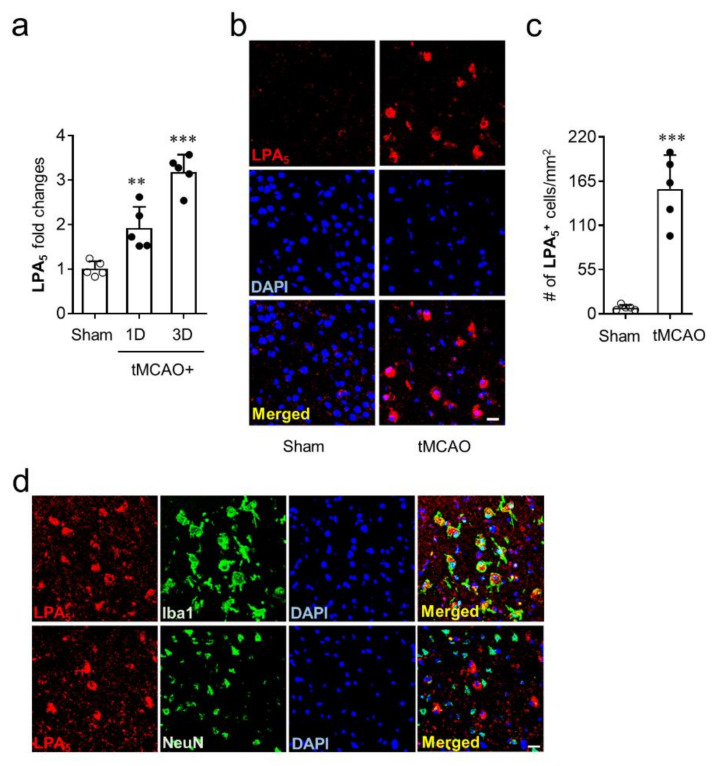
LPA_5_ is upregulated in the ischemic core region and its upregulation occurs in activated microglial after tMCAO challenge. Ipsilateral brain samples from sham and tMCAO-operated mice were used to determine expression levels of mRNA or protein and cellular localization of LPA_5_. (**a**) LPA_5_ expression at mRNA levels were determined at one and three days after tMCAO challenge by qRT-PCR analysis. (**b**,**c**) LPA_5_ expression at protein levels was determined in the core region at three days after tMCAO challenge by immunofluorescence. (**b**) Representative images for LPA_5_ expression. (**c**) Quantification of LPA_5_-immunopositive cells. (**d**) Cellular localization of upregulated LPA_5_ was determined in the ischemic core region at three days after tMCAO challenge by double immunofluorescence. Representative images for LPA_5_ expression on microglia (Iba1) or neuron (NeuN). Scale bars, 20 µm. *n* = 5 mice per group. ^**^ *p* < 0.01 and ^***^ *p* < 0.001 versus sham.

To identify whether LPA_5_ is upregulated in the brains of mice with tMCAO-induced transient focal cerebral ischemia, mRNA levels of LPA_5_ were determined at one and three days after tMCAO challenge. LPA_5_ mRNA expression was significantly upregulated at both one and three days after tMCAO challenge compared with the sham group (Figure 1a). Moreover, the mRNA level of LPA_5_ was higher at three days than it was at one day after tMCAO challenge (Figure 1a). This upregulation was reaffirmed at the protein levels using immunofluorescence. LPA_5_ protein was also upregulated in the post-ischemic brain at three days after tMCAO. LPA_5_-immunopositive cells were mainly observed in the ischemic core region, and their number was markedly increased in the post-ischemic brain compared with the sham group (Figure 1b,c). Next, we analyzed a type of cells that are responsible for LPA_5_ upregulation in the ischemic core region using double immunofluorescence for LPA_5_ with cell-specific markers, NeuN (neuron) or Iba1 (activated microglia). LPA_5_ was dominantly expressed on activated microglia but not on neurons (Figure 1d). These results demonstrate that LPA_5_ was upregulated in the post-ischemic brain, particularly in activated microglia that are known to play important roles in brain damage after ischemic challenge. In addition, these results strongly indicate that LPA_5_ may act as a pathogenic factor for ischemic stroke.

### 3.2. Suppressing LPA_5_ Activity Reduces Brain Damage in Mice with tMCAO Challenge

**Figure 2:**
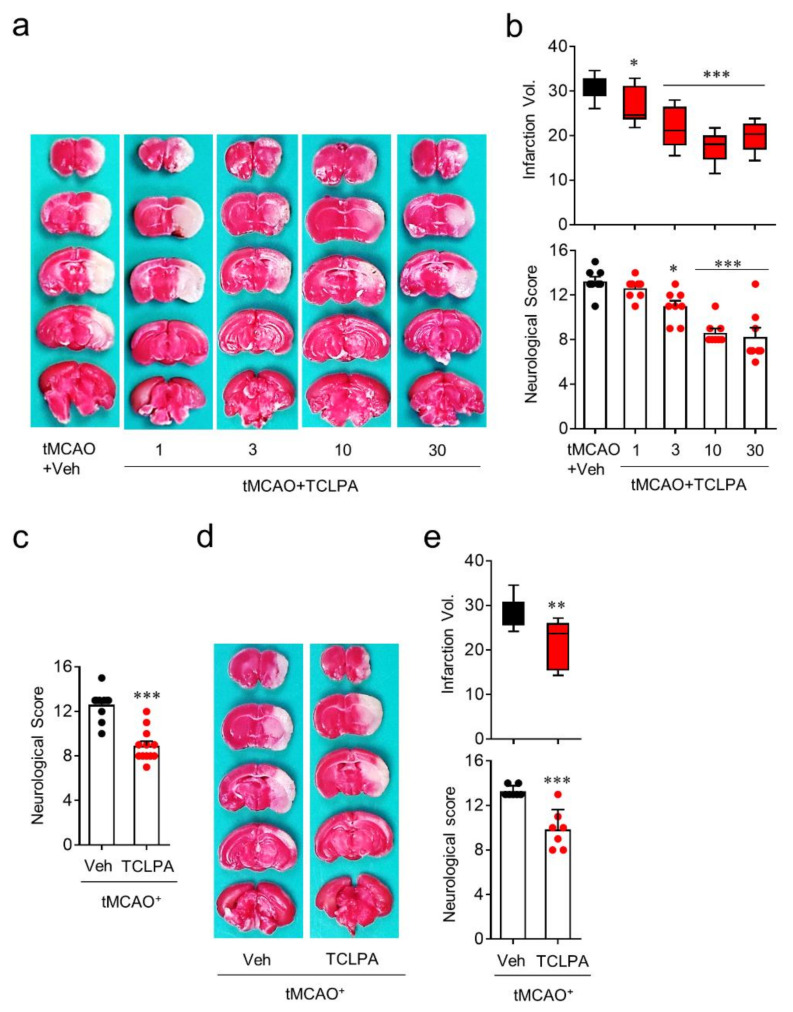
LPA_5_ antagonism attenuates ischemic brain damage after tMCAO challenge. Mice were challenged with tMCAO. TCLPA5 (TCLPA: 1, 3, 10, and 30 mg/kg, i.p.) was administered to mice immediately after reperfusion. Alternatively, 10 mg/kg TCLPA5 was administered to mice at three hours after reperfusion. (**a**,**b**) Effects of TCLPA5 administration (1, 3, 10, and 30 mg/kg, i.p., administered immediately after reperfusion) on infarction volume (**a**,**b**) and neurological deficit score (**b**) were assessed at one day after tMCAO. Representative images for TTC-stained brain sections (**a**) and quantification of brain infarction volume or neurological score indicating neurological functions (**b**). *n* = 8 mice per group. (**c**) Effects of TCLPA5 administration (10 mg/kg, i.p., administered immediately after reperfusion) on the neurological deficit score were assessed three days after tMCAO. *n* = 10 (tMCAO + Veh) and 12 (tMCAO + TCLPA). (**d**,**e**) Effects of delayed TCLPA5 administration (10 mg/kg, *i.p.*, administration at three hours after reperfusion) on infarction volume (**d**,**e**) and neurological deficit score (**e**) were assessed at one day after tMCAO. Representative images for TTC-stained brain sections (**d**) and quantification of brain infarction volume or neurological score (**e**). *n* = 7 mice per group. ^*^ *p* < 0.05, ^**^ *p* < 0.01, and ^***^ *p* < 0.001 versus vehicle-administered tMCAO group.

To identify whether LPA_5_ acts as a pathogenic factor for cerebral ischemia, we used an LPA_5_ antagonist, TCLPA5. To examine whether suppressing LPA_5_ activity by TCLPA5 administration would attenuate brain damage, mice were administered either vehicle or different doses of TCLPA5 (1, 3, 10, and 30 mg/kg, i.p.) immediately after reperfusion. Brain infarction was assessed by TTC staining and the neurological score was assessed by modified neurological severity scores (mNSS) at one day after tMCAO challenge. TCLPA5 administration significantly attenuated brain infarction (Figure 2a,b) and improved neurological deficit score (Figure 2b) in a dose-dependent manner in tMCAO-challenged mice compared to vehicle-administered mice. These neuroprotective effects of LPA_5_ antagonism were most pronounced with 10 mg/kg of TCLPA5, and this dosage was chosen for further in vivo experiments. Next, we determined whether the neuroprotective effects of LPA_5_ antagonism would persist up to three days after tMCAO challenge. TCLPA5 administration (10 mg/kg, i.p.) immediately after reperfusion significantly improved the neurological deficit score (Figure 2c), even at three days after tMCAO challenge. We further investigated the effects of delayed TCLPA5 administration. Even when TCLPA5 was administered to mice 3 h after reperfusion, brain infarction and the neurological deficit score were significantly attenuated (Figure 2d,e). These in vivo data demonstrate that suppressing LPA_5_ activity reduces brain damage in mice with ischemic stroke, further suggesting that LPA_5_ may be a therapeutic target to treat cerebral ischemia.

**Figure 3:**
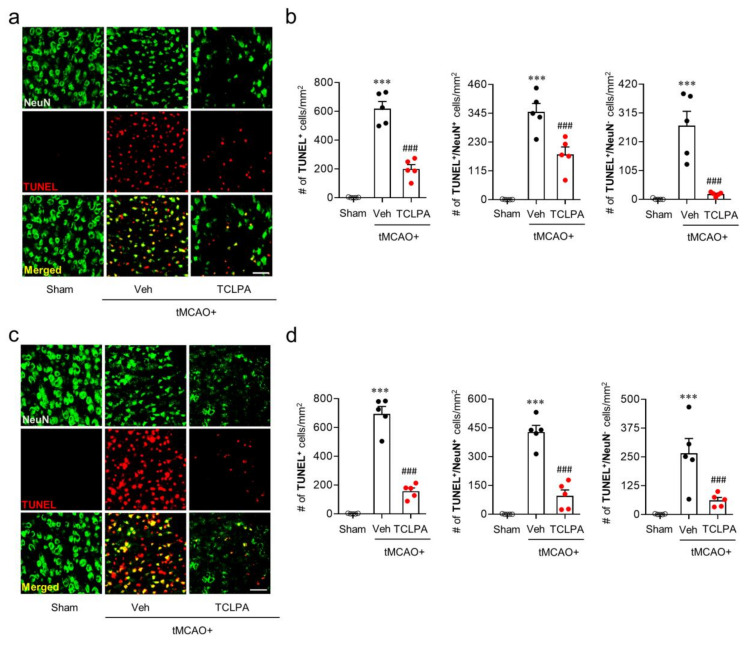
LPA_5_ antagonism attenuates neuronal and non-neuronal apoptosis in the injured brain after tMCAO challenge. Mice were challenged with tMCAO, and TCLPA5 (TCLPA: 10 mg/kg, i.p.) was administered immediately after reperfusion. Neuronal and non-neuronal apoptosis was determined by TUNEL assay at one and three days after tMCAO challenge. (**a**,**b**) Effects of TCLPA5 on neuronal and non-neuronal apoptosis were assessed at one day after tMCAO challenge. Representative images of TUNEL-positive and TUNEL/NeuN-double positive cells in the ischemic core region (**a**) and quantification (**b**). (**c**,**d**) Effects of TCLPA5 on neuronal and non-neuronal apoptosis were assessed at three days after tMCAO challenge. Representative images of TUNEL-positive and TUNEL/NeuN-double positive cells in the ischemic core region (**c**) and quantification (**d**). Scale bars, 50 µm. *n* = 5 mice per group. ^***^ *p* < 0.001 versus sham; ^###^ *p* < 0.001 vs. vehicle-administered tMCAO group.

It is known that many cells, including neurons, can undergo apoptosis in the post-ischemic brain [39,40]. To determine whether LPA_5_ is involved in neuronal and non-neuronal apoptosis after ischemic challenge, we analyzed the extent of neurodegeneration by TUNEL assay at one and three days after tMCAO challenge (Figure 3). In the vehicle-administered tMCAO group, marked numbers of neuronal and non-neuronal cells underwent apoptosis at both one (Figure 3a,b) and three days (Figure 3c,d) after tMCAO challenge, and this neuronal and non-neuronal death was significantly attenuated by suppressing LPA_5_ activity through TCLPA5 administration. These results indicate that LPA_5_ could be involved in cell apoptosis, particularly neuronal apoptosis in an injured brain after ischemic challenge.

### 3.3. Suppressing LPA_5_ Activity Attenuates Microglial Activation and Proliferation in Post-Ischemic Brain after tMCAO Challenge

**Figure 4:**
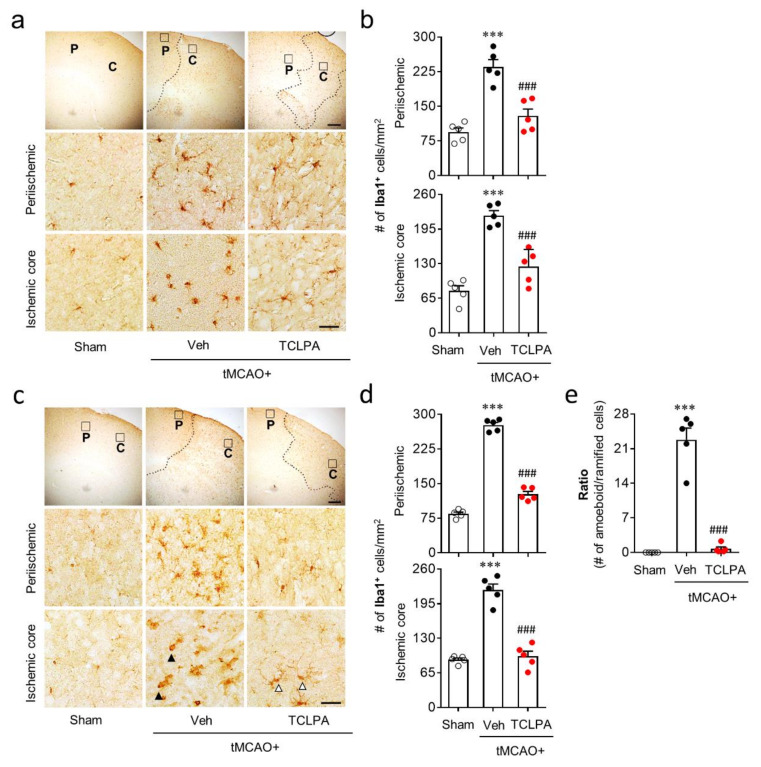
LPA_5_ antagonism attenuates microglial activation in the injured brain after tMCAO challenge. Mice were challenged with tMCAO. TCLPA5 (TCLPA: 10 mg/kg, i.p.) was administered immediately after reperfusion, and microglial activation was determined by Iba1 immunohistochemistry at one and three days after tMCAO challenge. (**a**,**b**) The effects of TCLPA5 on microglial activation were assessed one day after tMCAO challenge. (**a**) Representative images of Iba1-immunopositive cells in periischemic (“P”) and ischemic core (“C”) regions. Diagram boxes in upper panels display cerebral areas from which images in middle and bottom panels were obtained. Dotted lines separate periischemic and ischemic core regions. (**b**) Quantification of Iba1-immunopositive cells in each region. (**c–e**) Effects of TCLPA5 on microglial activation were assessed at three days after tMCAO challenge. Representative images of Iba1-immunopositive cells (**c**) and quantification of such cells (**d**) in each region. (**e**) The ratio of amoeboid to ramified Iba1-immunopostive cells in the ischemic core region. Open or closed arrowheads (in **c**) indicate ramified or amoeboid Iba1-immunopositive cells, respectively. Scale bars, 200 µm (top) and 50 µm (middle and bottom). *n* = 5 mice per group. ^***^ *p* < 0.001 versus sham; ^###^ *p* < 0.001 versus vehicle-administered tMCAO group.

Microglial activation is a well-known pathogenic feature in cerebral ischemia brains [25]. In the current study, we found that LPA_5_ upregulation mainly occurred in activated microglia in the post-ischemic brain (Figure 1). Therefore, LPA_5_ could be involved in microglial activation in the post-ischemic brain. To address this, we determined whether suppressing LPA_5_ activity through TCLPA5 administration would attenuate microglial activation. Iba1 immunoreactivities and morphological changes were determined to analyze microglial activation in the post-ischemic brain by time (one and three days after tMCAO challenge) and injured regions (the periischemic and the ischemic core regions). The numbers of Iba1-immunopositive cells were increased in both regions at one and three days after tMCAO challenge, and this effect was significantly attenuated by TCLPA5 administration (Figure 4a–d). In addition, it is well-known that ischemic challenge induces clear morphological changes of activated microglia from ramified to amoeboid cells in the ischemic core region at three days after tMCAO challenge [24,38,41]. We also observed a significant increase in amoeboid cells with Iba1 immunoreactivities in the ischemic core region at three days after tMCAO challenge (Figure 4e), which was markedly attenuated by TCLPA5 administration (Figure 4e). 

**Figure 5:**
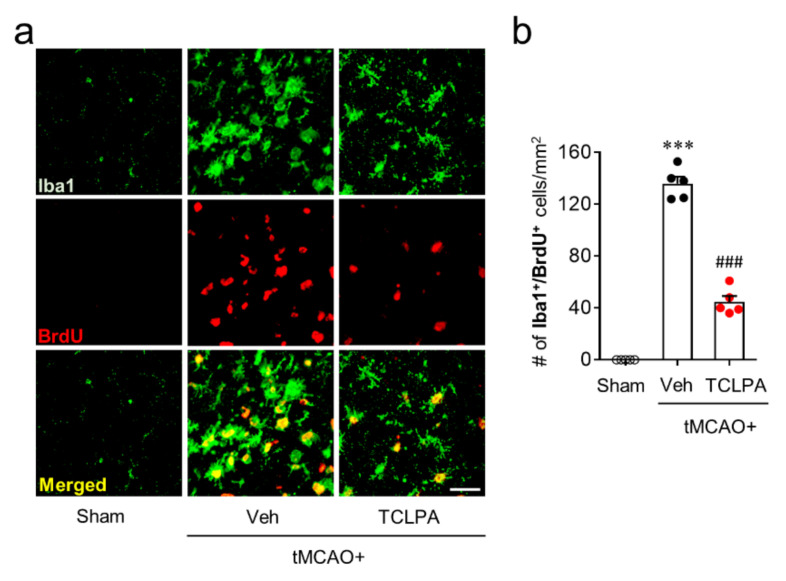
LPA_5_ antagonism attenuates microglial proliferation in injured brain after tMCAO challenge. Mice were challenged with tMCAO. TCLPA5 (TCLPA: 10 mg/kg, i.p.) was administered immediately after reperfusion, and microglia proliferation was assessed by Iba1/BrdU double immunofluorescence at three days after tMCAO challenge. (**a**). Representative images of Iba1/BrdU-double immunopositive cells in the penumbra region (the area between the periischemic and ischemic regions). Scale bar, 50 µm. (**b**) Quantification of Iba1/BrdU-double immunopositive cells. *n* = 5 mice per group. ^***^ *p* < 0.001 vs. sham; ^###^ *p* < 0.001 vs. vehicle-administered tMCAO group.

In addition to these features, activated microglia can proliferate in the marginal zone (the penumbra region: the area between the periischemic and the ischemic core regions) of the post-ischemic brain, which is another feature of microglial activation in cerebral ischemia [24,38]. Therefore, we also determined whether LPA_5_ could be involved in microglial proliferation in the penumbra region at three days after tMCAO challenge by BrdU/Iba1 double immunofluorescence. In the vehicle-treated group, the number of BrdU/Iba1 double-immunopositive cells was significantly increased in the penumbra region compared with the sham group (Figure 5a,b), demonstrating microglial proliferation. Suppressing LPA_5_ activity with TCLPA5 administration significantly reduced the number of BrdU/Iba1 double-immunopositive cells (Figure 5a,b), indicating that LPA_5_ could regulate microglial activation in the post-ischemic brain. Taken together, these data suggested that LPA_5_ may play a critical role in microglial activation in cerebral ischemia.

### 3.4. Suppressing LPA_5_ Activity Attenuates Upregulation of Proinflammatory Cytokines in Post-Ischemic Brain after tMCAO Challenge

**Figure 6:**
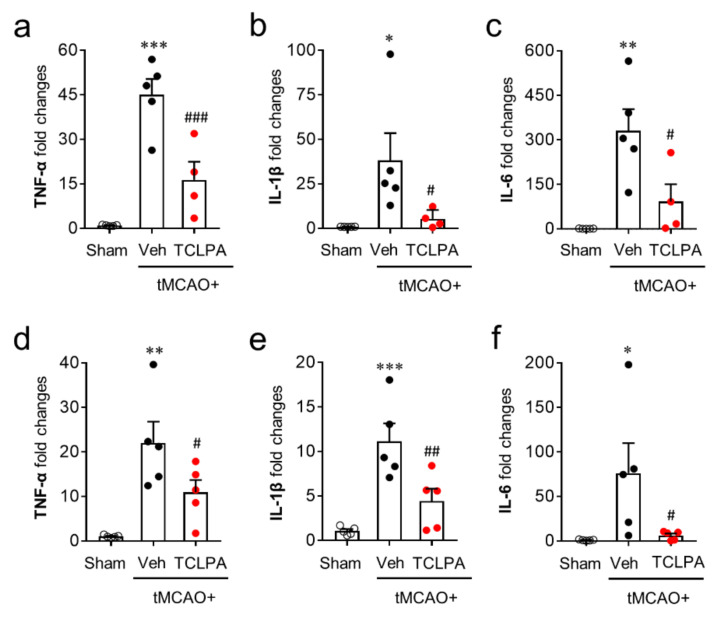
LPA_5_ antagonism attenuates mRNA expression levels of pro-inflammatory cytokines in the injured brain after tMCAO challenge. Mice were challenged with tMCAO. TCLPA5 (TCLPA: 10 mg/kg, i.p.) was administered immediately after reperfusion and mRNA expression levels of pro-inflammatory cytokines (TNF-α, IL-1β, and IL-6) were determined using qRT-PCR analysis at one (**a**–**c**) and three days (**d**–**f**) after tMCAO challenge. *n* = 4 ~ 5 mice per group. ^*^ *p* < 0.05, ^**^ *p* < 0.01, and ^***^ *p* < 0.001 versus sham; ^#^ *p* < 0.05, ^##^ *p* < 0.01, and ^###^ *p* < 0.001 versus vehicle-administered tMCAO group.

Activated microglia contribute to the productivities of pro- and anti-inflammatory cytokines in the post-ischemic brain, which is also a core pathogenic feature in cerebral ischemia [42,43]. To examine the roles of LPA_5_ in pro- and anti-inflammatory responses in the post-ischemic brain, we first determined temporal changes in the mRNA expression levels of pro-inflammatory cytokines (TNFα, IL-1β, and IL-6) in the post-ischemic brain at one and three days after tMCAO challenge. In the vehicle-treated group, the mRNA expression levels of pro-inflammatory cytokines were upregulated at both time points, and this upregulation was significantly attenuated by suppressing LPA_5_ activity (Figure 6a–f). 

**Figure 7:**
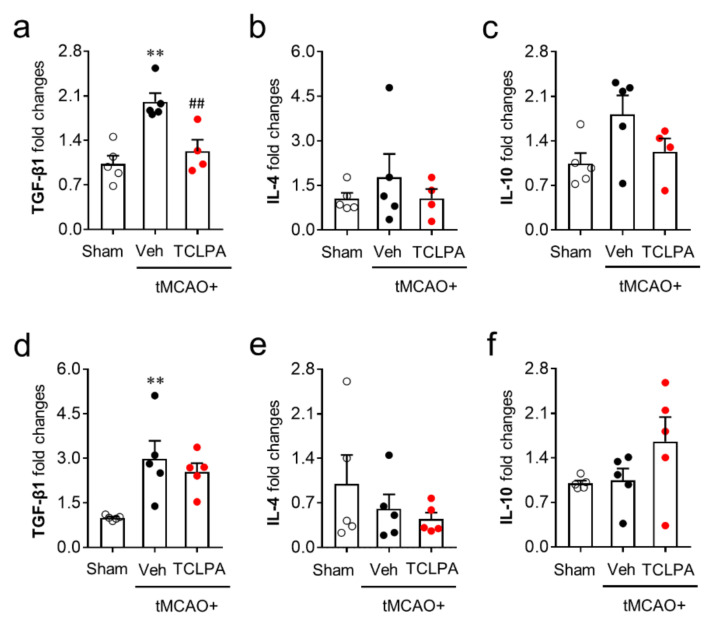
LPA_5_ antagonism does not increase mRNA expression levels of anti-inflammatory cytokines in the injured brain after tMCAO challenge. Mice were challenged with tMCAO. TCLPA5 (TCLPA: 10 mg/kg, i.p.) was administered immediately after reperfusion, and mRNA expression levels of anti-inflammatory cytokines (TGF-β1, IL-4, and IL-10) were determined by qRT-PCR analysis at one (**a**–**c**) and three days (**d**–**f**) after tMCAO challenge. *n* = 4 ~ 5 mice per group. ^**^ *p* < 0.01 versus sham; ^##^ *p* < 0.01 versus vehicle-administered tMCAO group.

We next determined whether LPA_5_ could also regulate anti-inflammatory responses by measuring the mRNA expression levels of anti-inflammatory cytokines (TGF-β1, IL-4, and IL-10) in the post-ischemic brain at both one and three days after tMCAO challenge. Unlike pro-inflammatory cytokines, the mRNA expression levels of most of the anti-inflammatory cytokines were not altered by suppressing LPA_5_ activity at both one and three days after tMCAO challenge (Figure 7). The marginal effects of TCLPA5 administration were only observed in TFG-β1 mRNA expression at just one day after the challenge (Figure 7a). These combined results clearly demonstrate that LPA_5_ can regulate mainly pro-inflammatory responses in the post-ischemic brain. 

**Figure 8:**
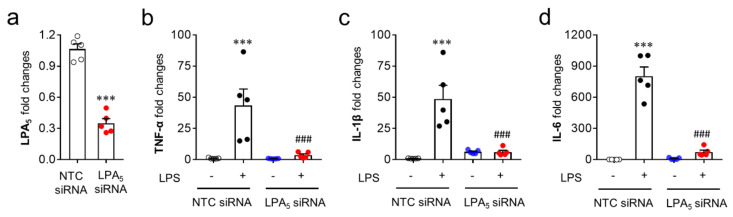
LPA_5_ knockdown attenuates mRNA expression levels of pro-inflammatory cytokines in LPS-stimulated BV2 microglia. Transfected BV2 microglia with non-target control (NTC siRNA) or LPA_5_ siRNA were stimulated with LPS (100 ng/mL) for 24 h and mRNA expression levels of LPA_5_ or pro-inflammatory cytokines (TNF-α, IL-1β, and IL-6) were then determined by qRT-PCR analysis. (**a**) Knockdown efficiency for LPA_5_. *n* = 5 per group. ^***^ *p* < 0.001 versus cells transfected with non-target control siRNA (NTC siRNA). (**b**–**d**) Effects of LPA_5_ knockdown on mRNA expression levels of TNF-α (**b**), IL-1β (**c**), and IL-6 (**d**). *n* = 5 per group. ^***^ *p* < 0.001 versus vehicle-treated cells with NTC siRNA; ^###^ *p* < 0.001 versus LPS-treated cells with NTC siRNA.

Since microglia are the cells responsible for pro-inflammatory responses in the post-ischemic brain, the modulatory role of LPA_5_ was reaffirmed in vitro using a BV2 microglial cell line stimulated with LPS, a well-known inducer of pro-inflammatory responses [44,45]. For this step, we used LPA_5_ siRNA instead of TCLPA5 because TCLPA5 exceeding 1 μM reduced cell viability in cells (our unpublished data). LPS exposure induced the upregulation of mRNA expression of pro-inflammatory cytokines (TNFα, IL-1β, and IL-6) in BV2 cells (Figure 8b–d), whereas LPA_5_ knockdown (Figure 8a) significantly reduced the mRNA expression levels of those cytokines (Figure 8b–d). These results indicate that LPA_5_ may be a critical molecule for pro-inflammatory responses in activated microglia.

## 4. Discussion

In this study, we identified LPA_5_ as a novel pathogenic factor in focal cerebral ischemia using a mouse model of transient focal cerebral ischemia. We demonstrated that LPA_5_ was upregulated in the post-ischemic brain, particularly in activated microglia. More importantly, we demonstrated that suppressing LPA_5_ activity attenuated brain damage, including brain infarction, functional neurological deficit, and neuronal cell death in mice with tMCAO challenge. Moreover, the present study demonstrated that LPA_5_ could be a critical factor for microglial activation, a well-known pathogenic event, in the post-ischemic brain. We also demonstrated that LPA_5_ could be associated with pro-inflammatory responses but not with anti-inflammatory responses in the post-ischemic brain. All these findings strongly support the claim that LPA_5_ is a pathogenic factor in cerebral ischemia.

In cerebral ischemia, LPA_1_ is the first LPA receptor subtype identified as a pathogenic factor. Pharmacological antagonism or genetic suppression of LPA_1_ activity has been shown to reduce brain damage such as brain infarction and functional neurological deficit and to attenuate pain responses in mice with tMCAO challenge [22,23,24]. Moreover, the pathogenic roles of LPA_1_ in cerebral ischemia have been associated with microglial activation and proinflammatory responses [24]. In the current study, we identified LPA_5_ as an additional receptor subtype that mediates the pathogenesis of cerebral ischemia with similar findings. However, it should be noted that LPA_5_ is upregulated in the post-ischemic brain because LPA_1_ was downregulated at mRNA expression levels in the post-ischemic brain [24]. It should also be noted that the number of activated microglia in the ischemic core region was reduced at three days after tMCAO challenge by suppressing LPA_5_ activity (the current study) but not LPA_1_ activity [24]. Interestingly, the current study demonstrates that upregulated LPA_5_ is mostly observed in activated microglia of the ischemic core region at three days after tMCAO challenge. This finding indicates that LPA_5_ may play a certain role in the ischemic core region, likely by regulating the numbers of activated microglia. However, even with these different results regarding LPA_1_, it should be noted that LPA_1_ is important for microglial activation in the ischemic core region at three days after tMCAO challenge. In fact, suppressing LPA_1_ activity reduced the soma size of Iba1-positive cells and the number of amoeboid cells in that region at three days after tMCAO challenge [24]. Therefore, both receptor subtypes should be considered to be regulators for microglial activation in the post-ischemic brain at both one and three days after tMCAO challenge. Regarding neuroinflammatory responses, these two receptor subtypes may lead to slightly different responses. It is clear that both LPA_1_ [24] and LPA_5_ (the current study) are involved in regulating pro-inflammatory responses in the post-ischemic brain, but they have slightly different effects on anti-inflammatory responses. In this study, LPA_5_ had no effect on the expression of anti-inflammatory cytokines in the post-ischemic brain at three days after tMCAO challenge. However, under the same condition, suppressing LPA_1_ activity has shown to increase IL-4 and decrease TGF-β1 at mRNA expression levels [24]. While these findings do not negate the importance of both LPA_1_ and LPA_5_ in cerebral ischemia in terms of pathogenesis, these two receptors do appear to play slightly different roles in areas injured by the ischemic challenge. 

It is well-known that neurons can undergo apoptosis after ischemic challenge. Besides neurons, other types of cells in the brain, such as microglia, astrocyte, endothelial cells, and infiltrating leukocytes, could also undergo apoptosis [46]. Apoptosis is the hallmark of ischemic-induced brain damage and the blockade of apoptotic processes can attenuate brain injury after ischemic challenge [47,48]. In this study, LPA_5_ was demonstrated to contribute to both neuronal apoptosis and non-neuronal apoptosis in the post-ischemic brain because suppressing LPA_5_ activity with TCLPA5 reduced cell apoptosis of both cases.

Microglial activation and their detrimental roles have been well noted in the pathogenesis of various CNS disorders, including spinal cord injury, trauma, and cerebral ischemia [13,25,49]. In cerebral ischemia specifically, microglia are robustly activated, as indicated by the increased number of activated cells, their proliferation, and their morphological transformation into neuroharmful amoeboid cells in the post-ischemic brain [24,38,50]. According to the results of the current and previous [24] studies, at least two LPA receptors (LPA_1_ and LPA_5_) can be considered as regulators of microglial activation in the post-ischemic brain. 

The roles of activated microglia in the post-ischemic brain are closely linked to pro- and anti-inflammatory responses, each of which induces secondary brain damage and drives neuroprotection in the post-ischemic brain [43,51,52]. In particular, proinflammatory cytokines can contribute to brain damage and long-term functional disability in the chronic phase of cerebral ischemia [53]. In contrast, anti-inflammatory cytokines can contribute to brain recovery or neuroprotection in the post-ischemic challenge [51]. These clearly contrasting phenomena could be used as a basic strategy to develop new therapeutics for cerebral ischemia, likely toward either the reduction of pro-inflammatory responses [54] or the enhancement of anti-inflammatory responses [51,55]. The currently identified roles of LPA_5_ were linked solely to pro-inflammatory responses after ischemic challenge: TCLPA5 administration attenuated the mRNA expression levels of pro-inflammatory cytokines (TNF-α, IL-1β, and IL-6) in the injured brain after tMCAO challenge, but failed to enhance the expression levels of anti-inflammatory cytokines (TGF-β1, IL-4, and IL-10). Instead, it only showed a marginal reduction in TGF-β1 mRNA at one day, but not at three days, after tMCAO challenge, and had no effect on other anti-inflammatory cytokines (IL-4 and IL-10). These findings indicate that LPA_5_ may be mainly associated with pro-inflammatory responses for disease progression rather than anti-inflammatory responses for neuroprotection in the injured brain after ischemic challenge. In line with our findings, studies using LPA_5_ antagonists have identified that the LPA/LPA_5_ signaling axis regulates pro-inflammatory response in vitro in BV2 microglia cell line or primary murine microglia [35,36,56]. LPA has been demonstrated to drive microglia toward cells with pro-inflammatory phenotype, including the upregulation of pro-inflammatory cytokines, but not toward those with anti-inflammatory phenotype [35]. The induction of pro-inflammatory phenotypes in LPA-stimulated BV2 cells or cultured microglia has been shown to be attenuated by TCLPA5 [35,36], AS2717638 [56], or compound 3 [56], all of which are LPA_5_ antagonists. Moreover, our in vitro data clearly demonstrated that LPA_5_ knockdown with its specific siRNA attenuated the mRNA upregulation of pro-inflammatory cytokines (TNF-α, IL-1β, and IL-6) in LPS-stimulated BV2 microglia. Taken together, these in vivo and in vitro results indicate that LPA_5_ could be a novel factor to regulate pro-inflammatory responses in the brain after cerebral ischemia. Considering the involvement of proinflammatory responses in the long-term disability of cerebral ischemia, these results further indicate that LPA_5_ antagonism might be able to exert long-term protective effects in cerebral ischemia.

In conclusion, we provided experimental evidence for a pathogenic role of LPA_5_ in the injured brain after ischemic challenge and showed that it regulated pathogenic events as well as neuroinflammatory responses, including microglial activation and pro-inflammatory responses. Recently, LPA receptors have been considered to be interesting targets for the development of new drugs in view of efforts to evaluate the clinical efficacies of LPA_1_ and LPA_1/3_ antagonists in tissue fibrosis (ClinicalTrials.gov ID: NCT01766817), psoriasis (ClinicalTrials.gov ID: NCT02763969), and systemic sclerosis (ClinicalTrials.gov ID: NCT01651143). Leading a successful case targeting LPA_5_ has also been shown to be feasible. The current study might provide insight into such a strategy, at least for cerebral ischemia. In fact, LPA_5_ antagonism immediately after reperfusion reduced brain damage, and this neuroprotective effect persisted up to three days after reperfusion. Moreover, delayed administration at three hours after reperfusion significantly reduced brain damage. To evaluate the potential effects of relevant treatments, it is believed to be crucial to know whether the beneficial effects of such treatments are maintained several days after ischemic challenge and whether they are maintained even by delayed treatment. In this view, the current study further indicates that LPA_5_ antagonism could be the basis of a strategy to develop therapeutics for treating cerebral ischemia.

## Figures and Tables

**Table 1:** Primer sets used for qRT-PCR analysis.

Gene	Forward	Reverse
*β-actin*	5′-AGCCTTCCTTCTTGGGTATG-3′	5′-CTTCTGCATCCTGTCAGCAA-3′
*TNF-α*	5′-CATCTTCTCAAAATTCGAGTGACAA-3′	5′-TGGGAGTAGACAAGGTACAACCC-3′
*IL-1β*	5′-CAACCAACAAGTGATATTCTCCATG-3′	5′-GATCCACACTCTCCAGCTGCA-3′
*IL-6*	5′-GAGGATACCACTCCCAACAGACC-3′	5′-AAGTGCATCATCGTTGTTCATACA-3′
*TGF-β1*	5′-CAACCCAGGTCCTTCCTAAA-3′	5′-GGAGAGCCCTGGATACCAAC-3′
*IL-4*	5′-GTCATCCTGCTCTTCTTTCTCG-3′	5′-TCTGTGGTGTTCTTCGTTGCT-3′
*IL-10*	5′-TGGCCTTGTAGACACCTTGG-3′	5′-AGCTGAAGACCCTCAGGATG-3′
*LPA_5_*	5′-AGGAAGAGCAACCGATCACA-3′	5′-ACCACCATATGCAAACGATG-3′
